# Neuromuscular Activation Patterns in Response to Windlass Stimulation and Biofeedback: A Surface EMG Study

**DOI:** 10.3390/sports14040158

**Published:** 2026-04-16

**Authors:** Laura Carrasco-Fernández, Álvaro Gómez-del Pino, Manuel García-Sillero, Pablo González-Cañizares, Jerónimo García-Romero, María Teresa Tomás, Javier Benítez-Porres

**Affiliations:** 1 Department of Human Physiology, Physical Education and Sport, Faculty of Medicine, University of Málaga, 29010 Málaga, Spainjeronimo@uma.es (J.G.-R.); 2 Department of Nursing and Podiatry, Faculty of Health Sciences, University of Málaga, 29010 Málaga, Spain; 3 Health & Technology Research Center, Escola Superior de Saúde de Lisboa, Instituto Politécnico de Lisboa, 1990-096 Lisboa, Portugal; 4 Internal Medicine UGC, Victoria Virgen University Hospital, Instituto de Investigación Biomédica de Málaga y Plataforma en Nanomedicina-IBIMA Plataforma BIONAND, 29010 Málaga, Spain; 5 CIBER Fisiopatología de la Obesidad y Nutrición (CIBEROBN), Instituto de Salud Carlos III, 28029 Madrid, Spain

**Keywords:** windlass mechanism, biofeedback, neuromuscular activation

## Abstract

Background: Handball involves unilateral, high-demand actions that increase injury risk. The Windlass mechanism (WM) is a position-dependent plantar fascia tensioning system, activated by dorsiflexion of the first metatarsophalangeal joint, which increases medial longitudinal arch stiffness and contributes to foot stability. WM activation can be mechanically simulated using hallux wedges to induce controlled dorsiflexion, allowing graded engagement of the mechanism under standardized conditions. The primary aim of this study was to investigate how different wedge inclinations, with and without visual biofeedback, affect foot muscle activity during squats in elite female handball players. Methods: Seventeen elite female handball players performed squats at 65% of one-repetition maximum under six conditions combining three wedge inclinations (0°, 10°, 30°) with the presence or absence of visual biofeedback. Electromyographic activity (RMS and %MVC) of intrinsic and extrinsic foot muscles was recorded. Results: A significant increase in left abductor hallucis activation with the 10° wedge without biofeedback. Visual biofeedback significantly increased RMS and %MVC in intrinsic foot muscles and increased RMS in the left gastrocnemius (*p* < 0.05). No significant interaction was observed between wedge inclination and biofeedback. Conclusions: Controlled activation of the WM via hallux wedges and the use of visual biofeedback modulate foot muscle activity during squats. These strategies may be considered in training programs aimed at improving foot stability and reducing injury risk in elite female handball players.

## 1. Introduction

Handball is a team sport characterized by high-intensity actions that impose complex and asymmetrical biomechanical demands on the musculoskeletal system [1]. This contact sport requires explosive and predominantly unilateral actions such as jumps, ball throws, constant changes in direction, turns, and accelerations, generating repetitive mechanical loading that may induce functional imbalances between limbs [2]. Specifically, the repeated execution of unilateral movements can lead to functional asymmetries between limbs, resulting in muscular imbalances that affect performance and increase the risk of injury [3].

Within this dynamic, the contralateral lower limb plays a fundamental role in postural stability and in initiating the kinetic chain during actions such as throwing, transferring forces toward the trunk and upper limb [4]. Recent studies have shown that significant imbalances between the dominant and non-dominant limbs can negatively impact key sports performance variables, such as strength and speed [5].

The foot is a biomechanically complex structure with multiple degrees of freedom, fundamental for stability, support, and load transmission during gait [6]. During the initial contact phase, the foot acts as a rigid platform that facilitates impact absorption, while in the mid-stance and propulsion phases, its adaptability and flexibility allow load attenuation and optimization of stability and locomotor efficiency [7]. From an experimental perspective, the Windlass mechanism can be mechanically stimulated through passive dorsiflexion of the hallux, which increases plantar fascia tension and promotes elevation of the medial longitudinal arch even under static conditions. The biomechanical coupling between first metatarsophalangeal joint dorsiflexion and arch deformation through plantar fascia tension has been well described in studies of human locomotion and foot mechanics [8]. This mechanism is activated by dorsiflexion of the first metatarsophalangeal joint (1st MTPJ), which generates tension in the plantar fascia, causing elevation of the medial longitudinal arch and increasing foot stiffness. This process transforms the foot into an efficient lever during the propulsion phase, optimizing the transfer of forces to more proximal segments [9].

The proper functioning of the WM is crucial not only for mechanical efficiency but also for injury prevention [10]. Alterations in its activation or in the mobility of the hallux joint can compromise foot stability and functionality, which has been linked to pathologies such as plantar fasciitis, medial arch collapse, hallux limitus, and hallux rigidus [11]. During the terminal stance phase, extension of the 1st MTPJ tensions the plantar aponeurosis, causing approximation between the hindfoot and forefoot, which facilitates arch elevation and promotes supination, a biomechanical condition necessary for effective propulsion [12]. If this mechanism is compromised, the foot may remain in prolonged pronation, reducing its functional capacity and increasing the load on proximal and distal structures [13].

Likewise, limitations in dorsiflexion of the 1st MTPJ, whether due to structural causes or improper footwear use, can hinder the proper activation of the WM, altering load distribution in the plantar arch and promoting the development of overuse injuries [14].

In addition to the aforementioned concept of the WM, which can be influenced by the inclusion of external elements (wedges), there are other neurophysiological factors that can lead to significant changes. One of the approaches that has shown the most evidence in this regard is biofeedback, particularly when used via EMG [15]. Its use has demonstrated changes in muscle excitability and has proven useful in recovery processes for lower limb injuries, facilitating healing [16]. Furthermore, these powerful changes have been observed in other muscle groups throughout the body [17], making it a proven tool for inducing changes in muscle behavior.

In the present study, wedges of different inclinations were positioned bilaterally under the head of the first metatarsophalangeal joint to induce controlled dorsiflexion and generate graded mechanical activation of the Windlass mechanism without requiring dynamic gait. This approach allowed the standardization of the mechanical stimulus across participants and experimental conditions, enabling the investigation of neuromuscular responses associated with different levels of Windlass engagement during a functional strength task such as the squat [18]. In this context, the lower limb musculature is classified into intrinsic muscles, associated with controlling the plantar arch and forefoot stability, and extrinsic muscles, responsible for force generation and propulsion [19]. Intrinsic muscles, such as the abductor hallucis, play a key role in WM activation, while extrinsic muscles, such as the gastrocnemius, contribute to power generation in activities like squatting and jumping [20].

To assess their functional performance, electromyographic variables such as Maximum Activation Level (%MVC), which reflects the peak muscle recruitment, and Root Mean Square (RMS), which indicates the average level of activation during dynamic or sustained tasks, are used [21]. Evaluating both metrics allows for the identification of differential responses to mechanical or sensory stimuli and guides preventive strategies as well as the optimization of neuromuscular performance [22].

Given the physical demands and biomechanical requirements inherent to handball, it is essential to consider both functional asymmetry between limbs and the proper functioning of the foot’s stabilizing mechanisms to optimize performance and reduce injury risk [23]. Although some studies have confirmed the benefits of KW and specific musculoskeletal foot exercises, there is no scientific evidence regarding the effects of using wedges with different inclinations under the 1st MTP, combined with biofeedback strategies, on neuromuscular performance during strength and power tasks. Therefore, the main objective of this study is to analyse the impact of varying activation levels of the WM and the application of biofeedback on force production and vertical power during squatting, fundamental motor pattern, in a sample of professional female handball players. This approach aims to provide applied evidence on the relationship between foot-ankle complex function and performance in key sport-specific actions, while also offering practical tools for injury prevention and training optimization.

## 2. Materials and Methods

### 2.1. Study Design

A quasi-experimental within-subjects design with repeated measures was employed. Participants were recruited through intentional, non-probabilistic, and convenience sampling. Female handball players participated voluntarily. The data collection period lasted four weeks, including two weeks of prior familiarization with the experimental procedures.

### 2.2. Population

A total of 17 elite female handball players participated (Aged: 29.88 ± 4.23, height: 170.11 ± 7.7 cm, BMI: 23.04 ± 1.72 kg·m^2^). The players were recruited from the Málaga Costa del Sol Women’s Handball Club, current European champions (EHF European Cup, 2021).

Regarding the inclusion criteria, players were selected based on their specific playing positions, as well as the dominance of their upper and lower limbs. All participants had advanced experience in strength training and demonstrated proper squat technique, which was verified by sports trainers. Additional variables included the one-repetition maximum (1RM) in the squat exercise and performance with a load corresponding to 65% of their 1RM.

It was observed that the dominant upper limb used for throwing was contralateral to the dominant lower limb used during the take-off phase of jumping, reflecting the asymmetrical nature of handball-specific movement patterns. Furthermore, each player’s history of sport-related injuries over the course of their professional career was recorded.

As for exclusion criteria, any player who had a current injury at the time of data collection or who had suffered a significant musculoskeletal injury in the six months prior, potentially affecting jump performance, was excluded from participation.

### 2.3. Process

The technical team of the Costa del Sol Women’s Handball Club (Málaga) team was contacted and they were informed of the entire procedure that would be carried out. The search protocol was carried out in accordance with the ethical guidelines of the Declaration of Helsinki [24] and was approved by the ethics committee at the University of Málaga (code: 38-2019-H). The subjects were informed of the conditions of the study and were aware of the possible risks of the experiment and signed an informed consent form pledging their willingness to participate.

**Figure 1:**
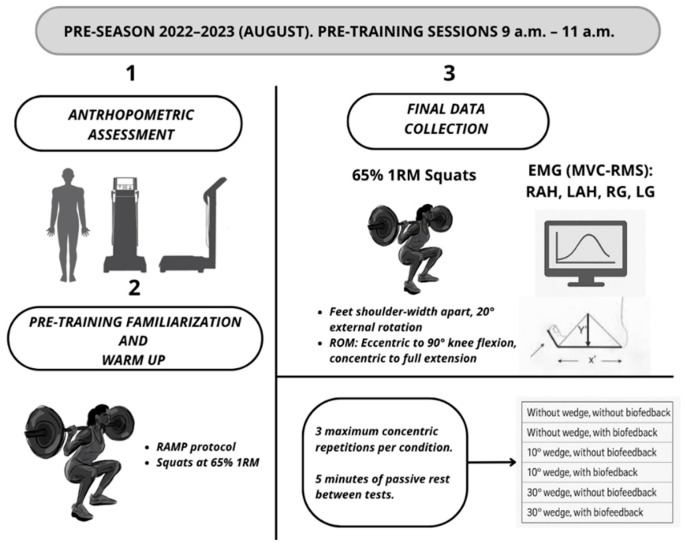
Organization of data collection. RAMP (Raise, Activate, Mobilize, Potentiate); ROM (range of motion); EMG (Electromyography); %MVC (Maximum Activation Level); RMS (Root Mean Square); RAH (Right abductor hallucis); LAH (Left abductor hallucis); RG (Right gastrocnemius); LG (Left gastrocnemius).

All measurements and tests were carried out in the laboratory of effort physiology and sport medicine at the University of Malaga and at laboratory of Eshmun Sport Clinic in Malaga. The assessments were conducted during the preseason (August 2022–2023), in the morning (9–11 a.m.), prior to the training session and after a minimum period of 12 h without strenuous physical activity (Figure 1).

### 2.4. Data Collection Techniques and Instruments

#### 2.4.1. Body Composition

For the analysis of body composition, body weight was previously measured with a scale (SECA, Hamburg, Germany) and body height using a wall-mounted stadiometer (Holtain Ltd., Crymych, UK).

Body mass index (BMI) was calculated by dividing the participant’s body weight in kilograms by the square of their height in meters. Muscle mass, fat mass, and body composition (% fat, % muscle) were assessed using the Inbody^®^ 770 model (Inbody 770, Inbody, Seoul, Republic of Korea).

#### 2.4.2. Warm-Up and Familiarization

A structured warm-up protocol was applied following the RAMP model (Raise, Activate, Mobilize, Potentiate) [25], aiming to optimize neuromuscular readiness and reduce injury risk. The protocol began with a progressive increase in core temperature and heart rate, followed by activation of the main muscle groups and mobilization of the key joints involved. The final phase consisted of potentiation exercises specific to the squat movement pattern, ending with progressive isotonic squats performed on Element + Multipower (Technogym Trade Company 2017, Cesena, Italy). Subsequently, a familiarization session with the main exercise was conducted, involving squats at 65% of the one-repetition maximum (1RM) to ensure proper technique execution and adaptation to the load stimulus. This load was selected as it represents a moderate intensity, sufficient to elicit neuromuscular activation and motor adaptation while minimizing the risk of fatigue or injury. Previous studies have recommended using 60–70% of 1RM during familiarization sessions to optimize movement technique and intermuscular coordination, particularly in intra-subject experimental designs [26].

#### 2.4.3. Squat Protocol and Biofeedback

The back squat was employed as the primary exercise, performed at 65% of the estimated one-repetition maximum (1RM) [26]. The initial stance consisted of feet positioned shoulder-width apart, with approximately 20° of external rotation. The movement involved a controlled eccentric phase reaching 90° of knee flexion (thighs parallel to the floor), followed by a concentric phase to full leg extension. Three maximal concentric repetitions were completed for each experimental condition, with five minutes of passive rest between sets to minimize fatigue [27]. Six conditions were examined, resulting from the combination of two biofeedback modalities (present vs. absent) and three wedge inclinations (0°, 10°, and 30°): (1) no wedge without biofeedback, (2) no wedge with biofeedback, (3) 10° wedge without biofeedback, (4) 10° wedge with biofeedback, (5) 30° wedge without biofeedback, and (6) 30° wedge with biofeedback. The six experimental conditions (3 wedge angles × 2 biofeedback conditions) were presented in a randomized order for each participant to minimize potential order and fatigue effects. The sequence of conditions was randomized individually using a randomization procedure prior to the experimental session. Adequate rest periods were provided between conditions to ensure consistent performance across all trials.

The wedges were designed using CAD-CAM technology and manufactured from high-density EVA material, measuring 9 × 2 × 4 cm, with inclinations of 10° and 30°, respectively. They were placed bilaterally under the head of the hallux to induce dorsiflexion at the metatarsophalangeal joint (MTPJ) and promote activation of the windlass mechanism. The 10° wedge simulated dorsiflexion typical of the propulsive phase of gait, while the 30° wedge represented the maximum functional range reported in the literature [28].

Biofeedback was applied only in the assigned conditions and included real-time visual feedback of the muscle activation curve provided by the electromyograph software. The curve was presented as a continuous line graph, allowing participants to monitor and adjust their contractions during the exercise. Additionally, verbal cues were provided by the evaluator to ensure maximal effort during the concentric phase. This combination of visual and verbal feedback allowed precise neuromuscular control and facilitated the assessment of biofeedback effects on muscle activation [29].

#### 2.4.4. Surface EMG Protocol

The surface electromyographic (EMG) activity during voluntary contractions was recorded using the wireless mDurance system (mDurance Solutions SL, Granada, Spain) with a sampling rate of 2000 Hz, following the SENIAM protocol guidelines for bipolar surface electrode placement [30]. Intrinsic foot muscles, specifically the right (RAH) and left (LAH) abductor hallucis, as well as extrinsic muscles including the right (RG) and left (LG) medial gastrocnemius, were assessed during squat execution and isometric contractions.

**Figure 2:**
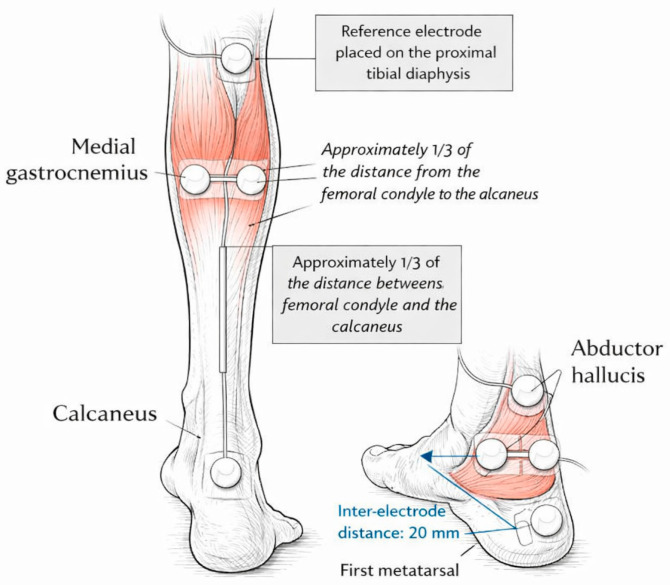
Surface EMG sensor placement on the medial gastrocnemius and abductor hallucis.

Prior to recording, the skin was prepared by cleaning with alcohol to reduce impedance and improve electrical conductivity. Pre-gelled Ag/AgCl electrodes (10 mm diameter, 1 mm thickness, 20 mm inter-electrode distance) were placed over the muscle bellies of the right and left medial gastrocnemius and over the tendons of the right and left abductor hallucis along the medial border of the foot, just posterior to the head of the first metatarsal, oriented along the muscle fibers from the tendon insertion at the first toe region toward the calcaneus (Figure 2). Electrodes were secured with transparent adhesive tape to prevent displacement. A reference electrode was positioned on the proximal tibial diaphysis [31]. Electrode size, placement, and careful skin preparation were chosen to minimize crosstalk from adjacent muscles.

For EMG signal normalization, unilateral 30 s maximum voluntary contractions (MVCs) were performed: maximal plantar extension on a leg press for the medial gastrocnemius, and maximal isometric plantarflexion combined with abduction of the first toe in standing position for the abductor hallucis. This allowed normalization of EMG activity for each muscle and limb independently.

During the experimental phase, EMG activity was recorded during squats performed under different conditions: without wedges and with 10° and 30° wedges, and with or without visual and auditory biofeedback.

The RMS variable was used to quantify the average intensity of the EMG signal and assess muscle activation levels, measured throughout the entire concentric phase of the 3 maximum repetitions in each set. while the MVC (% of maximal voluntary contraction) variable measured the maximal force consciously generated by the muscle [32]. Signals were filtered using a fourth-order Butterworth bandpass filter with a cut-off frequency at 20–450 Hz and smoothed (0.025 s RMS window, 0.0125 s overlap). Basal sEMG levels were checked before each session, and recordings were synchronized with repetitions and start/end positions using a position transducer [31].

### 2.5. Statistical Analysis

A repeated measures ANOVA design 3 × 2 was employed for data analysis, with two within-subject factors: KW angle (0°, 10°, and 30°) and biofeedback condition (with and without visual feedback). This model was applied to each dependent variable derived from surface electromyography: %MVC and RMS for each analyzed muscle (RAH, LAH, RG, LG). The sphericity assumption was tested using Mauchly’s test, and when violated, the Greenhouse-Geisser correction was applied to adjust the degrees of freedom in the ANOVA.

In the presence of significant main effects or interactions, post hoc pairwise comparisons were performed with Bonferroni correction to control for Type I error. When the assumptions of normality and homogeneity were not met, non-parametric alternative tests were used: specifically, the Friedman test for comparing multiple related conditions and the Wilcoxon signed-rank test for pairwise contrasts in related samples. In addition to the *p*-value, effect sizes were reported to complement the clinical and practical interpretation of the findings. Partial eta squared (η^2^ partial) was used for main effects in the ANOVA, while the coefficient r (r = z/√n) was calculated for post hoc comparisons and non-parametric tests.

Statistical analyses were performed using IBM SPSS Statistics v30.0 (IBM Corp., Armonk, NY, USA). The significance level was set at *p* < 0.05, although values in the range 0.05 < *p* < 0.10 were also reported as statistical trends, considering the exploratory nature of the study and the moderate sample size.

## 3. Results

The sample size (n = 17) provided statistical power greater than 80% to detect a medium effect size (f = 0.25) in a repeated measures design with 6 conditions (3 × 2), at a significance level of 0.05, according to calculations performed using G*Power v3.1.

Table 1 presents descriptive statistics for physical parameters and total body composition. Among the participants, 14 had the left leg as their dominant leg for jumping, while only 3 had the right leg as dominant (82.35% vs. 17.65%).

A specific within-subject analysis was performed to compare the three no-biofeedback conditions regarding neuromuscular activation (MVC and RMS) of the analyzed muscles (Table 2), aiming to evaluate the pure effect of mechanical activation of the WM induced by wedges at different angles (0°, 10°, 30°).

Maximum Activation Level (%MVC) without biofeedback: Among the no-biofeedback conditions, only the LAH muscle showed significant differences in %MVC (Friedman test: *p* < 0.05), while RAH, RG, and LG did not exhibit relevant changes (*p* > 0.05). Post hoc analyses (Wilcoxon) for LAH revealed statistically significant differences only between W0° and W10° (*p* = 0.0056, r = 0.72), with the effect of W10° without biofeedback being greater than the rest of the conditions.

Root Mean Square activation (RMS) without biofeedback: RMS results also showed significant differences only in the LAH muscle across no-biofeedback conditions (Friedman test: χ^2^ = 13.06, *p* = 0.0015). Post hoc analyses (Wilcoxon) for LAH revealed statistically significant differences between W0° and W10° (*p* = 0.0021, r = 0.706) and between W10° and W30° (*p* = 0.0110, r = 0.603), with the effect of W10° without biofeedback being greater than the rest of the conditions. The other muscle groups (RAH, RG, LG) did not show significant differences between conditions without biofeedback (*p* > 0.05).

Regardless of the wedge conditions, Table 3 presents the comparisons of muscle activity between biofeedback and no-biofeedback conditions. The presence of biofeedback resulted in statistically significant increases in RMS in the intrinsic muscle LAH (*p* < 0.05), followed by significant increases in %MVC activation in the intrinsic muscles RAH and LAH (*p* < 0.05). Regarding the extrinsic muscles, biofeedback significantly increased RMS only in the LG muscle (*p* < 0.05).

**Figure 3:**
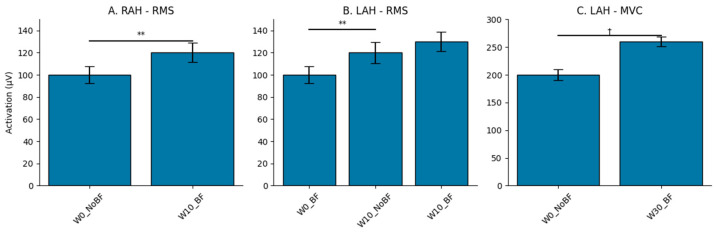
Comparison of muscle activation between conditions with and without biofeedback. Note: Comparison of muscle activation between wedge conditions (W0°, W10°, W30°) with and without biofeedback (BF vs. No BF). *p*-values and significant post hoc comparisons are reported for Maximum Activation Level (%MVC) and Root Mean Square (RMS) activation in microvolts (µV) in intrinsic foot muscles (LAH): Left Abductor Hallucis), *p* < 0.01 (**), and ^†^ indicates trend toward significance (*p* < 0.1).

Figure 3 provides a comprehensive comparison of muscle activation, assessed through %MVC and RMS, between conditions with and without biofeedback, across varying wedge angles (0°, 10°, and 30°).

%MVC with Biofeedback: Significant differences in %MVC were observed for RAH and LAH when using biofeedback without wedges (*p* < 0.05, r > 0.49). Combining wedges and biofeedback produced a greater effect on MVC, particularly in RAH (*p* = 0.0021, r = 0.71), while LAH showed a positive trend that did not reach significance (*p* = 0.0887). No significant differences were found for RG and LG (*p* > 0.05).

Mean Activation (RMS) with Biofeedback: Conditions W10°_NoBF and W30°_NoBF elicited the highest RMS values in RAH and LAH, with large effect sizes (r = 0.81 and 0.75 for RAH; r = 0.775 and 0.534 for LAH). Biofeedback without wedges (W0°_BF) increased RMS in LAH compared to W0°_NoBF, approaching significance (*p* = 0.0569, r = 0.465). When combining wedges and biofeedback (W10°_BF, W30°_BF), no additional RMS increases were observed (*p* > 0.05). For extrinsic muscles, only LG showed an isolated RMS increase with W30°_NoBF.

## 4. Discussion

The primary objective of this study was to examine the effects of WM activation, elicited through passive hallux dorsiflexion using wedges at varying inclinations (0°, 10°, and 30°) in combination with the presence or absence of biofeedback, on the neuromuscular activity of intrinsic (RAH, LAH) and extrinsic (RG, LG) foot muscles during both %MVC and submaximal (RMS) conditions.

The results indicate that both the mechanical intervention and the application of biofeedback significantly modulated the activation levels of intrinsic foot muscles. Moreover, although the effects on extrinsic musculature were less pronounced, biofeedback and mechanical loading produced clinically meaningful responses, particularly in the gastrocnemius, where RMS in the left medial gastrocnemius without biofeedback increased with the 30° wedge compared to 10° and 0° wedges.

In this study, a progressive increase in muscle activation was observed, particularly in the RMS values of the intrinsic foot muscles RAH and LAH, as passive dorsiflexion of the hallux was increased using 10° and 30° wedges compared to the 0° This pattern is consistent with the activation of the Windlass mechanism (WM), which has been described as fundamental for the elevation of the medial longitudinal arch and the stabilization of the foot during the propulsion phase of gait [19].

These findings are consistent with previous research demonstrating enhanced intrinsic foot muscle activity during passive hallux dorsiflexion, attributed to functional shortening of the plantar fascia and increased tension in stabilizing structures [10].

This effect was more pronounced in RMS measurements, as RMS is a more sensitive indicator of sustained muscle activation during submaximal or postural tasks, such as those induced by hallux positioning. In contrast, MVC reflects maximal voluntary effort, which may not be as affected by passive mechanical modifications of the environment [32].

However, in certain cases such as the LAH muscle, activation recorded with a 30° wedge was lower than that observed with a 10° wedge. This finding may result from neuromuscular inhibition associated with muscle over-lengthening, which can impair contractile efficiency [33]. Additionally, excessive dorsiflexion may generate pathological stress in soft tissues or induce neuromuscular fatigue, thereby limiting fine motor control [34]. Taken together, these findings suggest that the combination of moderate mechanical stimulation, through controlled activation of the Windlass mechanism, and visual biofeedback may promote enhanced neuromuscular activation of the intrinsic foot musculature. This interpretation is supported by previous studies demonstrating that both mechanical loading and EMG-based biofeedback independently contribute to increased muscle activation and improved neuromuscular control [15]. This finding is clinically relevant, emphasizing the importance of calibrating mechanical stimuli to optimize muscle activation without inducing negative compensatory mechanisms.

Regarding the extrinsic musculature, specifically the right and left medial gastrocnemius (RG and LG), no significant changes in neuromuscular activation were observed across experimental conditions in MVC and RMS (*p* > 0.05), except for the LG muscle in RMS without biofeedback. In this case, relevant differences were observed between the 30° wedge and the other conditions. This finding is consistent with previous research indicating that the gastrocnemius primarily responds to dynamic tasks involving force and power generation such as walking, running, or jumping and is less sensitive to localized manipulations or low-intensity stimuli under static or postural conditions [35]. This differential response may be attributed to the specific biomechanical role of extrinsic muscles, which are structurally adapted for rapid, high-intensity contractions and gross motor function [27]. In contrast, intrinsic foot muscles serve a specialized role in fine postural adjustments and medial arch stabilization during low-impact activities [36]. Due to their anatomical proximity to the foot’s bony structures and their involvement in local sensorimotor control, these muscles exhibit heightened neuromuscular sensitivity to subtle mechanical inputs and fine motor modulation, which is reflected in significant variations in RMS and MVC values even under static or low-demand conditions [37].

Evidence that visual biofeedback significantly increases activation of intrinsic foot muscles, even in the absence of mechanical stimuli such as wedges, underscores its potential as a valuable tool in proprioceptive and neuromuscular training [38]. Proprioceptive training aims to enhance body awareness, sensory perception, and fine motor control, which are key components for dynamic stabilization and injury prevention [29]. By providing real-time feedback on muscle activation, visual biofeedback facilitates more effective motor learning, promoting selective and adaptive recruitment of intrinsic foot muscles, which are essential for maintaining the medial longitudinal arch and postural stability [39].

In the context of neuromuscular rehabilitation and training, the incorporation of visual biofeedback can optimize the nervous system’s ability to modulate muscle activation, thereby improving strength, coordination, and postural control. This is particularly relevant in athletes with proprioceptive deficits or impaired motor control, where enhanced activation and control of intrinsic foot muscles through biofeedback may translate into significant functional benefits, such as improved load absorption and prevention of injuries related to foot instability [40].

A particularly notable finding was that the highest activation levels were recorded in the left foot, especially in the LAH muscle. RMS and MVC values were highest at W10°, compared to W0° and W30°. These values represent comparisons among conditions within the same muscle. Since most participants reported the left lower limb as dominant for performing the jump, this pattern may reflect greater neuromuscular efficiency, refined motor control, and motor familiarity on the dominant side [41]. Lateral dominance has been associated with structural and functional differences at both cortical and peripheral levels, leading to asymmetries in muscle activation during specific tasks [42]. From an applied perspective, these findings suggest that the dominant limb may exhibit a greater capacity to respond effectively to training stimuli, whether through visual biofeedback or mechanical manipulations, resulting in enhanced muscle recruitment and postural control. This increased neuromuscular efficiency could be attributed to motor familiarity and specific adaptations arising from preferential use of the dominant side [43]. Therefore, in training and rehabilitation programs, it is essential to consider limb dominance to personalize interventions, placing special emphasis on the activation and strengthening of the non-dominant limb. Such strategies may help improve muscular and functional symmetry, thereby reducing the risk of injuries related to neuromuscular imbalances and optimizing athletic performance [44]. Furthermore, optimizing neuromuscular control in both limbs is key to preventing foot and lower extremity pathologies, promoting joint stability and biomechanical efficiency during dynamic tasks and postural stabilization [45].

In summary, the findings of this study provide a deeper understanding of how activation of the Windlass mechanism and the incorporation of visual biofeedback can modulate neuromuscular activation of the intrinsic foot muscles, highlighting the key role of limb dominance in this response. The identification of a potential optimal range of mechanical stimulation and the positive impact of biofeedback open new avenues for designing personalized interventions aimed at enhancing fine motor control and joint stability.

These results hold particular relevance for rehabilitation and sports training, where improvements in muscle recruitment and correction of neuromuscular asymmetries may translate into a reduced incidence of injuries and enhanced functional performance. Given the growing interest that specific strength training in this anatomical area is having in the field of sports, we consider these findings to be highly relevant to the daily lives of many athletes [6].

However, given the complex interplay between mechanical, neuromuscular, and functional factors, further research involving dynamic tasks and clinical populations is warranted to validate and extend these findings. The knowledge generated by this study encourages a reconsideration of traditional training and rehabilitation strategies, advocating for the integration of tools such as biofeedback and targeted mechanical modulations to optimize muscle activation and promote functional symmetry across limbs.

The main limitation of this study was the small sample size, which limits the generalizability of the results and the analysis of differences based on player positions. The menstrual cycle was not considered, which could affect some physiological variables.

Although body mass index (BMI), body fat percentage, and muscle mass were recorded, these variables were not included as covariates in the statistical analysis, as they were not part of the primary objective of the study. However, given their potential influence on EMG signals, particularly through subcutaneous tissue thickness, they should be considered in future research.

Leg dominance was not specifically accounted for in the analysis, which may influence neuromuscular activation patterns and should be considered in future studies. Although the squat involves joint movement, the activation of the foot intrinsic muscles and gastrocnemius was assessed during predominantly isometric phases, providing a static component for these muscles and allowing analysis of their neuromuscular control under controlled conditions. These results should be complemented with studies involving more dynamic activities. Furthermore, the wedge intervention was acute, so long-term effects and adaptations are unknown.

## 5. Conclusions

The combination of moderate mechanical stimulation (10° wedge) and visual biofeedback may enhance neuromuscular activation of the intrinsic foot musculature. Precise calibration of stimulus intensity appears to be essential to avoid potential adverse effects and to maximize effectiveness in rehabilitation and training contexts.

Visual biofeedback in the absence of mechanical stimulation may also serve as an effective tool during early or complementary training phases, facilitating motor learning and selective activation of intrinsic muscles. The dominant lower limb demonstrated greater efficiency in muscle recruitment and improved motor control in response to stimuli such as visual biofeedback and mechanical stimulation.

It is recommended that coaches and rehabilitation professionals in female handball incorporate specific programs integrating visual biofeedback and controlled mechanical stimuli, alongside proprioceptive and unilateral exercises designed to replicate sport-specific demands. These interventions can optimize medial longitudinal arch stability, correct muscular imbalances, and reduce injury risk, thereby contributing to improved athletic performance.

## Figures and Tables

**Table 1:** Descriptive statistics of physical parameters and total body composition.

Variable	Mean ± SD
Age (years)	29.88 ± 4.23
Height (m)	1.70 ± 0.07
Weight (kg)	67.21 ± 8.92
BMI	23.04 ± 1.72
Muscle Mass (kg)	29.71 ± 3.20
Fat Mass (kg)	14.42 ± 4.92
Body Fat (%)	20.83 ± 4.51
1RM (kg)	137.12 ± 19.09
65% 1RM (kg)	89.13 ± 12.41

Note: Data is shown as mean and standard deviation (SD).

**Table 2:** Comparison of Muscle Activity Under No Biofeedback Conditions.

Muscle	Variable	W0°	W10°	W30°	χ^2^ (Friedman)	*p*-Value	Post Hoc Comparisons (*p*-Value)	Effect Size (r)	Best Condition
RAH	MVC (µV)	285.4 ± 140.2	412.6 ± 210.3	398.7 ± 195.6	4.59	0.101	W0° vs. W10°: 0.18; W0° vs. W30°: 0.12; W10° vs. W30°: 0.35	0.30; 0.35; 0.20	No significant differences
RAH	RMS (µV)	142.3 ± 92.5	165.8 ± 101.4	158.6 ± 97.2	4.59	0.101	W0° vs. W10°: 0.22; W0° vs. W30°: 0.15; W10° vs. W30°: 0.40	0.28; 0.33; 0.18	No significant differences
LAH	MVC (µV)	210.7 ± 120.6	340.5 ± 180.2	298.4 ± 165.1	7.41	0.025 *	W0° vs. W10°: 0.0056 **; W0° vs. W30°: 0.089; W10° vs. W30°: 0.58	0.72; 0.42; 0.12	W10° > W0°
LAH	RMS (µV)	120.4 ± 85.3	198.6 ± 110.7	170.2 ± 102.8	13.06	0.0015 ***	W0° vs. W10°: 0.0021 **; W0° vs. W30°: 0.078; W10° vs. W30°: 0.011 **	0.71; 0.40; 0.60	W10° > W0° > W30°
RG	MVC (µV)	310.2 ± 200.5	520.3 ± 310.6	495.1 ± 290.4	0.82	0.662	W0° vs. W10°: 0.80; W0° vs. W30°: 0.75; W10° vs. W30°: 0.90	0.10; 0.12; 0.08	No significant differences
RG	RMS (µV)	180.5 ± 130.2	220.4 ± 150.7	210.6 ± 140.9	2.24	0.327	W0° vs. W10°: 0.50; W0° vs. W30°: 0.48; W10° vs. W30°: 0.60	0.22; 0.20; 0.18	No significant differences

Note: Friedman test and post hoc pairwise comparisons of muscle activation variables reported for Maximum Activation Level (MVC) and Root Mean Square (RMS) activation across no biofeedback conditions (W0°, W10°, W30°), in intrinsic foot muscles (RAH): Right Abductor Hallucis; (LAH): Left Abductor Hallucis) and extrinsic muscles (RG: Right gastrocnemius; LG: left gastrocnemius). *p*-values from Friedman tests are reported along with pairwise post hoc comparisons (Wilcoxon signed-rank tests). Effect sizes (r) are included for each comparison. Significant differences are indicated as *p* < 0.05 (*), *p* < 0.01 (**), and *p* < 0.001 (***).

**Table 3:** Muscle activity between biofeedback and no-biofeedback conditions, without considering the effect of wedge angles.

Muscle	Variable	No Biofeedback (Mean ± SD)	Biofeedback (Mean ± SD)	*p*-Value	Z	Effect Size (r)	Clinical Interpretation
RAH	MVC (µV)	312.6 ± 185.4	368.9 ± 210.7	0.027 *	−2.201	0.534	Biofeedback ↑ MVC
RAH	RMS (µV)	145.2 ± 98.6	160.3 ± 105.1	0.3289	−1.018	0.247	No significant
LAH	MVC (µV)	228.4 ± 140.8	301.7 ± 175.2	0.040 *	−2.059	0.499	Biofeedback ↑ MVC
LAH	RMS (µV)	130.1 ± 90.4	185.9 ± 115.6	0.0093 **	−2.533	0.614	Biofeedback ↑ RMS
RG	MVC (µV)	402.7 ± 260.5	465.8 ± 290.3	0.263	−1.160	0.281	No significant
RG	RMS (µV)	190.6 ± 140.2	215.4 ± 155.8	0.3060	−1.065	0.258	No significant
LG	MVC (µV)	355.2 ± 220.6	420.3 ± 250.1	0.207	−1.302	0.316	No significant
LG	RMS (µV)	165.8 ± 120.3	210.7 ± 135.9	0.0448 *	−2.012	0.488	Biofeedback ↑ RMS

Note: Comparison of muscle activation between with and without biofeedback (BF vs. No BF) using paired Wilcoxon tests. *p*-values, Z statistics, and effect sizes (r) are reported for Maximum Activation Level (%MVC) and Root Mean Square (RMS) activation in intrinsic foot muscles (RAH): Right Abductor Hallucis; (LAH): Left Abductor Hallucis) and extrinsic muscles (RG: Right gastrocnemius; LG: left gastrocnemius) *p* < 0.05 (*), *p* < 0.01 (**), and 0.05 < *p* < 0.10 (statistical trends).

## Data Availability

The data supporting the findings of this study are openly available in the RIUMA institutional repository of the University of Málaga at https://hdl.handle.net/10630/45402 (accessed on 12 February 2026).

## Abbreviations

WM	Windlass mechanism
1st MTPJ	First metatarsophalangeal joint
KW	Kinetic wedge
MVC	Maximum Voluntary Contraction
RMS	Root Mean Square
1RM	One-repetition maximum
BMI	Body mass index
RAMP	Raise, activate, mobilize, potentiate
EMG	Electromyographic
RAH	Right abductor hallucis
LAH	Left abductor hallucis
RG	Right medial gastrocnemius
LG	Left medial gastronecmius
